# Peer support in an outpatient clinic for people living with human immunodeficiency virus: a qualitative study of service users’ experiences

**DOI:** 10.1186/s12913-022-07958-8

**Published:** 2022-04-25

**Authors:** Anita Øgård-Repål, Rigmor C. Berg, Vegard Skogen, Mariann Fossum

**Affiliations:** 1 Centre for Caring Research- Southern Norway, Department of Health and Nursing Science, University of Agder, Grimstad, Aust-Agder, Norway; 2 Divison of Health Services, Norwegian Institute of Public Health, Oslo, Norway; 3 Faculty of Health Sciences, UiT The Arctic University of Norway, Tromsø, Norway; 4 Department of Infectious Diseases, Medical Clinic, University Hospital of North Norway, Tromsø, Norway

**Keywords:** HIV, Peer support, Outpatient clinics, Social support, In-depth interviews, Directed content analysis

## Abstract

**Background:**

Although human immunodeficiency virus (HIV) has become a manageable condition with increasing life expectancy, people living with HIV (PLHIV) are still often isolated from society due to stigma and discrimination. Peer support provides one avenue for increased social support. Given the limited research on peer support from the perspective of PLHIV, this study explored their experiences of peer support organised by healthcare professionals in an outpatient clinical setting.

**Methods:**

The study used a qualitative, descriptive research design for an in-depth understanding of peer support provided to PLHIV in the context of outpatient clinics. Healthcare professionals contributed to the recruitment of 16 participants. We conducted in-depth interviews about participants’ experiences of peer support, and performed a directed content analysis of the data. Further, we sorted the data into pre-determined categories.

**Results:**

The pre-determined categories constituted attachment, social integration, an opportunity for nurturance, reassurance of worth, reliable alliance, and guidance. The identified themes were: *gained emotional support*, *disclosure behaviour allowed garnering of emotional support*, *non-disclosure promoted the need to meet a peer*, *experienced a sense of belonging*, *activated an opportunity for mutual support*, *means to re-establish belief in one’s own worth*, *perceived a positive affirmation of disease management*, *facilitated dialogue about disease management*, the outpatient clinic as *a safe place*, *and a setting for flexible, individualised support*.

**Conclusions:**

This study highlights the peer support experiences of PLHIV in the context of outpatient clinics. The participants’ experiences align with previous findings, showing that peer support contributes to mutual emotional support between peers. This is particularly important in cultures of non-disclosure where PLHIV experience intersectional stigma. Additionally, our results show outpatient clinics to be supportive surroundings for facilitating peer support, ensuring confidentiality in peer support outreach. Therefore, peer support contributes positively to individualising outpatient clinic services to meet the changing needs of PLHIV.

**Supplementary Information:**

The online version contains supplementary material available at 10.1186/s12913-022-07958-8.

## Background

For over 25 million people living with human immunodeficiency virus (PLHIV) with access to antiretroviral therapy (ART), their life expectancy is approaching that of the general population [1, 2]. However, human immunodeficiency virus (HIV) is a chronic lifelong condition (CLLC) [3], involving complex needs with an increased burden of non-communicable diseases and mental health disorders [4–6]. In addition, PLHIV report poorer health-related quality of life than the general population [7, 8]. This may stem from negative societal reactions towards PLHIV, defining HIV as one of the most stigmatised diseases in almost every culture worldwide [9–12]. Being subjected to societal prejudice and stigma negatively affects emotional well-being of PLHIV. PLHIV often constitute members of marginalised groups, such as sexual minorities and people who use intravenous drugs; thus, many experience intersectional stigma [13, 14].

As a result of many PLHIV becoming disconnected from society [9, 15], with their multidimensional concerns being followed by a need for confidentiality, their ability to reach out for help is negatively affected. Consequently, the degree of social support is impacted [16, 17]. This is unfortunate given the recognised relationship between social support and health [15]. Nevertheless, social support can be a potential source of resilience when PLHIV experience stress, for example, in response to the stigma connected to HIV [18–20]. Specifically, peer support for PLHIV seems to be a crucial resource, as it has been found to increase social support and reduce HIV-related stigma [21, 22].

Peer support, which refers to the support provided by a peer who has had similar personal experiences, has increasingly become a recognised outreach for PLHIV. It strengthens supportive resources in healthcare services, increases self-management, and supports PLHIV in taking an active role in self-management of a CLLC in daily life [23–25]. Notably, the involvement of users in their healthcare services may contribute to increased empowerment and promote a person-centred service that is sensitive and responsive to emotional well-being [3, 26, 27]. Therefore, peer support aligns with the World Health Organization’s (WHO) strategy that calls for a person-centred chronic care for PLHIV [27, 28]. WHO defines individualised peer support as ‘one-to-one support provided by a peer who has personal experiences of issues and challenges like those of another peer who would like to benefit from this experience and support’ [29, p.1]. Different peer support models have been applied across various healthcare contexts. These range from informal visits and sharing experiences to formal appointments focused on practical information sharing [23, 30, 31].

The effectiveness of a range of peer support interventions for PLHIV has recently been reviewed. According to Berg et al.’s systematic review [25], peer support improves ART adherence, reduces the risk of virologic failure, improves viral suppression, and increases long-term retention in care. In addition, other research findings indicate that peer support provides an opportunity for individuals to be an active part of their recovery process, is flexible enough to be applied to varied settings, and is responsive to people’s varied needs [24, 25, 32].

Although the effectiveness of a range of peer support interventions has been studied, a recent review [33] demonstrated a scarcity of studies that explored experiences with peer support from the receiver’s perspective. The results of the review indicated multiple benefits of meeting a peer supporter, necessitating a clarification of the peer support provided to PLHIV as a CLLC. In addition, although we are aware that different contexts can affect the contribution of peer support, there is limited knowledge about the incorporation of peer supporters as an integral part of healthcare services in outpatient clinics (OPCs) [25]. Therefore, the objective of this study was to explore how PLHIV experience the support provided by peers in OPCs.

### Theoretical frameworks

Although this study specifically focused on a peer support program as a part of healthcare services, peer support offers services beyond the traditional medical model of care. Several researchers clarify the concept of peer support in line with its varied contributions, including providing inspiration toward living a full life [23, 24, 31]. As a complement to general healthcare services, there is a recognition that peer support contributes to meeting needs at the individual level covering several dimensions of well-being [34]. The correlation between health and social support has been recognised in recent years [18]. Disclosure of their HIV status allows PLHIV to garner the social support they need [17]. Social support is associated with decreased anxiety and depression, and higher resilience, particularly pertaining to HIV-related stigma [18–20]. However, PLHIV often experience decreased social support following diagnosis [20].

Social support can serve several functions; Weiss [35] provides theoretical formulations for several purposes of social support. Although Weiss’s model originates from the context of loneliness, it captures important elements when conceptualising social support. He identifies six different social functions or ‘provisions’ needed to feel supported, thereby avoiding loneliness. The themes reflect what the participants gain from relationships with others.

First, guidance and reliable alliance are the most relevant functions to direct problem-solving in stressful situations. Second, the provision of reassurance of worth is related to others recognising one’s competence, skills, and values. Third, an opportunity for nurturance points to an essential aspect of feeling needed by others in interpersonal relationships. While this provision is not strictly considered social support, it indicates that giving and receiving in an interpersonal relationship may enhance health. This value is also recognised by Borkman [36], a leading researcher on the mutual support dynamic, as an essential component of peer support. The last functions described by Weiss are attachment and social integration. These functions regard the presence of affectional ties. Affectional ties concern emotional closeness to others that contribute to a sense of security. In contrast, social integration involves the feeling of belonging to a group that shares the same interests, concerns, and activities [35, 37].

## Methods

### Study design

This study used a qualitative, descriptive research design involving directed content analysis, which explores a phenomenon guided by existing theory [38, 39]. In-depth interviews were conducted to explore the qualitative, lived experience of meeting a peer supporter [40, 41]. We also examined several aspects related to living with HIV. Using a qualitative method, this study provided comprehensive data on the phenomenon, as it allowed an assessment of both similar and different components of peer meetings [42].

### The advisory group

Two user representatives of PLHIV, one non-governmental organisation representative, one nurse, and one medical doctor, were invited to form an advisory group. The purpose of the advisory group was to secure lay community experts’ perspectives and feedback throughout the research process, and thus improve the quality of the research. The nurse and the medical doctor worked at separate HIV OPCs. Among the user representatives were men, women, an immigrant, and a member of a sexual minority group. The advisory group clarified terms, explored research questions, developed the interview guides with the research team, and was actively involved in the data analysis. To decrease the risk of potential cooptation of peer support values in the meetings related to power dynamics between the members of the adivsory group, we conducted separate, independent meetings with the PLHIV representatives.

### Study setting

The HIV OPCs in Norwegian hospitals are funded by the government and part of the specialist healthcare services, and meet every person newly diagnosed with HIV at least once. As a national Norwegian standard, OPCs located in hospitals provide free medical follow-up and treatment of people infected with HIV [43]. When people are diagnosed with HIV, during their first consultation at the OPC, they meet an infectious disease specialist. Further, the OPCs provide regular follow-ups in general once to twice a year. Supplementary follow-ups are performed in collaboration with the primary healthcare and other parts of the specialist healthcare system depending on the patients’ needs, for example, mental and somatic comorbidity [43]. At the end of 2020, Norway had a low prevalence of 6,778 people diagnosed with HIV (4585 men and 2193 women) [42], and has achieved the United Nations Programme on HIV/AIDS 90–90-90 treatment targets developed in 2013. The 90–90-90 targets aim for 90% of all people with HIV knowing their status, 90% receiving sustained antiretroviral therapy, and 90% of people with HIV receiving ART having viral suppression [44, 45].

The setting for the peer program (described below) was five public OPCs situated in local hospitals in the four regional health authorities in Norway, two of which were university hospitals. The five OPCs provide the routine follow-up as described above. Until now, per support has only been offered to PLHIV through non-governmental organisations. The non-governmental organisations are situated in the larger cities in Norway, and thereby only available for one of the OPCs included in this study.

#### The peer support program

A user-initiated peer support program for PLHIV started nearly ten years ago as part of the healthcare services at one user-driven OPC serving PLHIV. A committee of PLHIV developed goals for healthcare services based on their needs and experiences. One goal was to establish peer support. This was because a peer supporter could offer assistance, grounded on values of equality, and thus an opportunity to focus the support on the direct, here-and-now needs with which the service users presented [46, 47]. As a result of the user-involvement process, five OPCs incorporated the peer support program as part of their healthcare services for PLHIV during 2019 and 2020. Healthcare professionals (HPs) at the five OPCs aim to provide peer support to the PLHIV enrolled at the respective OPC through a peer support program. HPs organise meetings between peers. Peer supporters work as independent consultants, and receive a payment (72 USD per consultation funded by the OPCs) as compensation for their contribution and coverage of travel expenses. The HPs provide the peer supporters with regular supervision. In addition, the peer supporters regularly meet for peer discussions and assessments.

Peer supporters are PLHIV, receiving treatment and care at one of the included OPCs, and formally trained to be peer supporters through a training program jointly developed by the HPs and supporters. The non-peer-reviewed literature of Bloomsbury Patient Network, the UK’s National Training Program of Peer Mentors, *Project 100*, and National Standards for HIV Peer Support [21] inspired the training program and its implementation in OPCs. Inspired by the peer support training conducted in the UK [21], the peer support program's implementation and training were conducted in a dialogue between peer supporters and healthcare professionals at the different clinics to ensure that the values of peer support were understood and implemented.

### Recruitment strategy and eligibility criteria

We aimed to explore diverse levels of involvement, thoughts, and perceptions, to gain a thorough, in-depth understanding of the peer support experiences of PLHIV [43]. The HPs at the OPCs therefore purposively recruited PLHIV enrolled in the clinics who they believed could share valuable and rich experiences [42, 48].

The following eligibility criteria were used for PLHIV: 1) living with HIV, 2) enrolled in HIV clinical care at one of the OPCs, 3) aged 18 or older, 4) willing to sign written informed consent for study participation, and 5) having attended at least one peer support meeting. The participants could participate regardless of literacy, but they had to understand Norwegian or English. Individuals enrolled in an OPC were eligible irrespective of whether they were receiving ART.

The number of participants to be interviewed was considered after reading through three initial interview transcripts and initiating preliminary coding. We aimed for an iterative, context-dependent decision regarding sample size to reach data saturation. Through the analytical process with predefined categories, the 16 interviews provided us with an increasingly comprehensive picture of the predefined categories as well as an ability to develop sub-categories. Following Malterud’s guidance of sample size [49], and considering the narrow study aim, quality of the interview data and the HPs’ involvement in participant recruitment, we found 16 interviews to have yielded sufficient information.

All 16 invited individuals agreed to participate. We covered the participants’ travel expenses and provided light refreshments during the interviews.

#### Data construction

The first author conducted face-to-face, in-depth, semi-structured interviews at participants’ convenience during spring and autumn of 2020. The interviews were conducted in office at the respective OPCs. The first author, who had not met any of the participants before, informed them that she was a registered nurse with prior interviewing experience. The first author made field notes immediately after each of the 16 interviews, which lasted between 30 and 60 min, with an average of 47 min. The interviews were audiotaped and transcribed verbatim. The participants were asked if they wanted to read the transcripts, but all of them declined the offer.

The current study formed part of a larger PhD study where a scoping review of the empirical literature on peer support for PLHIV was conducted. The results and patterns in the scoping review informed the interview guide of the current study. In addition, the interview guide was not pilot tested, but developed jointly by the authors and the advisory group. The advisory group contributed to the clarification of concepts based on relevance. It included 21 open-ended questions (Additional File 1).

### Analysis

In accordance with the description by Assarroudi et al. [36] and Hsieh and Shannon [37], we conducted a directed, qualitative content analysis to prepare, organise, and report the findings (see Additional File 2). Our directed content analysis was based on existing theory of the phenomenon [39, 42], namely social support.

First, the first and last author deductively applied Weiss’ six identified provisions of social relations as pre-determined categories: attachment, social integration, the opportunity for nurturance, reassurance of worth, reliable alliance, and guidance [35, 37, 50]. Then, the first and last author used an inductive process to develop specific codes within each pre-determined category [39].

The initial phase involved familiarisation with the textual data; the first and last author read through the transcripts to get a sense of the entire collected information. In the second stage, the data were de-identified and imported into the NVivo 12 software program to assist in coding and analysing the qualitative data. Next, we applied the pre-determined categories to the textual data, and the first author searched for meaningful units related to each of the pre-determined categories. Data found to be relevant, but not fitting into one of the pre-determined categories, inductively formed a new category. Finally, the first and last author coded the interviews according to the categorisation matrix defined by the coding rules, exemplified through sample quotes (see Additional File 3) [38].

In the next stage, meaningful units relating to each pre-determined category were inductively condensed by the first author. The first and last authors discussed the condensation. In stage four, the first author coded the condensed meaningful units and discussed the codes with the authors and the advisory group. The coding included reverting to the text and reanalysing to identify texts missing from the pre-determined categories [38]. Next, all authors examined the codes for differences and similarities and abstracted them into sub-categories in a back-and-forth process (see Table 1).

**Table 1:** Examples of the directed content analysis

*Meaningful units*	*Condensations*	*Codes*	*Sub-categories*	*Pre-determined categories*
‘I got support here at the hospital, and this is like my ‘health family’, talking to the nurse and the peer supporters. That is important’. (Cries when saying this) (P1)	Talking to nurses and peer supporters when needing support related to HIV	The hospital as a supportive family	Gaining emotional support	Attachment
‘It was good. I am not alone. I knew I was not alone, but I knew no one else’. (P3)	Meeting peer supporters provided a feeling of not being alone	Meeting peers promotes the feeling of not being alone	Experiencing a sense of belonging	Social integration
‘You have to be discreet all the time. I survive by being so quiet about this. I am happy that we had this peer talk here at the hospital. It is a typical problem that you really have to talk to someone about, but you cannot talk about it because people probably cannot relate, and they might be discriminating.’ (P4)	Need of discretion when afraid of being stigmatised; the hospital is the only place to meet peers	Non-disclosure of PLHIV prevents them from meeting other peers outside of the hospital	A safe place	OPCs as the setting for peer support

Finally, the sub-categories were abstracted into their representative pre-determined categories. The sub-themes were reviewed by members of the research team (AØ-R, RB, VS, and MF) before proceeding to the reporting phase. Any disagreements were discussed until a consensus was reached.

### Ethical considerations

The Norwegian Social Science Data Service approved of this study (NSD; reference number 184248). Information about the study was communicated both orally and in writing before the participants chose to participate. Informed consent was obtained from all participants before data collection started. Informed consent included information about participants’ opportunity to withdraw from the study at any time without negative consequences regarding their relationship with HPs at the OPCs. The manuscript preparation adhered to the Criteria for Reporting Qualitative Research (COREQ) checklist [51].

## Results

We interviewed six women and ten men, with ages ranging between 30 and 58 years (mean age: 44 years), representing characteristics for PLHIV in Norway. The findings concerning how the participants experienced peer support organised and undertaken by the OPCs were reported according to the pre-determined categories from the primary sources of provisions of social relationships. These constitute attachment, social integration, an opportunity for nurturance, reassurance of worth, reliable alliance, and guidance [35, 37]. Reliable alliance is, in this context, operationalised as ‘serving as a liaison between patients and clinical care, motivating patients to communicate and assert themselves to obtain regular and quality care, helping to identify local resources when needed’ (see Additional file 3). However, we found no meaningful units concerning the peer supporters providing support aligned with the ‘reliable alliance’ provision, although HPs offered this to our participants. Therefore, this provision was excluded from the results section. In addition, the category ‘OPCs as the setting for peer support’ was developed inductively (see categories in Table 2).

**Table 2:** Overview of the pre-determined categories and sub-categories

Support provided by peer supporters to PLHIV					
Pre-determined categories					
Attachment	Social integration	Opportunity for nurturance	Reassurance of worth	Guidance	OPCs as the setting for peer support
Sub-categories					
Gained emotional support	Non-disclosure promoted the need to meet a peer with similar concerns	Activated an opportunity for mutual support	Means to re-establish belief in one’s own worth	Perceived positive affirmation of disease management	A safe place
Disclosure behaviour allowed garnering of emotional support	Experienced a sense of belonging			Facilitated dialogue about disease management	A setting for flexible, individualised support

### Attachment

The participants expressed that they gained emotional support from peer supporters when they were short of other emotionally close relationships, or when their former close relationships were negatively affected or destroyed due to their HIV diagnosis. Conversely, participants who had disclosed their diagnosis to others, followed by a supportive response, did not get emotionally attached to the peer supporters.

#### Gained emotional support

Non-disclosure behaviour seemed to prevent participants from garnering emotional support from friends and family. They even recognised that they could not expect support when they did not disclose their HIV diagnosis:

‘I have no one to talk to. So, you go all by yourself. I have not said anything about my HIV to my friends, so I do not know whether they would support me or not’. (P11)

Some of the participants, who were immigrants and had experienced stigma related to HIV in their home country, said that when they disclosed their HIV diagnosis to their families, it was followed by rejection:

‘He [my father] told me I am a whore. My family had this perception irrespective of the amount of education they received. This is due to their culture and society. Mom said the same thing. It was such a bomb, such an electric current in my brain, so uncomfortable. Therefore, I just cut the phone. They disapproved of me’. (P10)

These participants described experiences of either non-disclosure behaviour or rejection when disclosing their diagnosis, which promoted a need to garner emotional support from other sources as a ‘substitute’ for the help generally received from friends and family. Thereby, some participants expressed that people connected to the OPCs were a ‘supportive family’. In addition, some of the participants had not disclosed their diagnosis to anyone outside of the hospital, in the sense that only the HPs and peer supporters knew about their HIV status.

#### Disclosure behaviour allowed garnering of emotional support

Some of the participants, mainly gay men with a Norwegian background who had chosen to disclose their HIV diagnosis to their family and/or friends, expressed emotional support from their close relations as a response:

‘I chose to share the diagnosis right away. I received no negative reactions. There may have been some worries at home, but that is how it will be. Thus, it has been the reaction I expected. I never thought there was going to be a problem at home or with close family and friends at all’. (P13)

It seems like the need for emotional support from peers living with HIV was reduced when participants disclosed their HIV diagnosis; the most important thing for them was to have someone to rely on when the need arose:

‘When it comes to friends, there are not so many questions... They ask if everything is okay, but there is no such thing as feeling sorry for me, which is the most important thing. This is not what I want. I have someone to talk to who can listen. This is often what you need... to get things off your chest, and I get that support’. (P13)

If a shortage of knowledge characterised the support they received from friends and family, the participants found their concerns and lack of rejection as an expression of support:

‘They are as supportive as you might expect them to be. HIV is no issue. When HIV is the pertinent topic, they are as supportive as one might expect them to be, considering the naivety of heterosexual adults, because they have very little knowledge’. (P12)

### Social integration

When non-disclosure increased the feeling of being alone, the participants found that peer supporters could provide them with a sense of belonging to a group with similar concerns.

#### Non-disclosure promoted the need to meet a peer

The participants described several reasons for their non-disclosure behaviour, followed by a need for support from peer supporters. They expressed a combination of protecting the family from being worried and protecting themselves. Some of the participants feared social and family exclusion if they were to share their HIV diagnosis:

‘I do not want to share my illness with them. We have a great relationship as a family. I do not want them to be afraid of me’. (P1)

Further, some participants explained their non-disclosure behaviour as a personal protection and response to experienced societal prejudices:

‘When it comes to HIV, it is like their reaction is that of disgust and fear. It is not an inspiration for disclosure, I must say. Thus, I am glad I am not open about having HIV’. (P12)

The participants’ non-disclosure behaviour promoted the need to meet other people with similar experiences and concerns:

‘When I need to talk, I call. I will call, and then I will come and talk if I need to’. (P1)

Some participants actively chose not to disclose their diagnosis to family and friends, preferring to avoid potential adverse reactions:

‘So, I do not know if I should be stigmatised. But I am afraid I will. For now, those who know have not reacted like that… But of course, I decide whom I disclose my diagnosis to’. (P6)

Thereby, participants asked for peer supporters to have someone to talk to about their HIV status. This seemed to be a way to address their HIV-related concerns.

#### Experienced a sense of belonging

The participants found that peer support left them with a sense of belonging to a group just by being present, as an immediate embodied feeling of togetherness, indicating that they affected the participants’ well-being:

‘It was good. I am not alone. I knew I was not alone, but I knew no one else. So really, meeting someone was...’(P3)

The results also indicated that the sharing of recognisable experiences and emotions created a supportive environment. The mutual disclosure between peers embraced the sharing of reflections, wonder, and engagement. The mutuality revealed itself as felt, lived, and true to the individuals involved.

‘We sat there and talked about our experiences, and then it coincided. We live in the same cultural context. And it was a bit like coming home’. (P12)

A meeting between peers became a place to openly share their worries, knowing that they would receive support for their emotions related to living with HIV. Peer supporters validated the participants’ experiences:

‘It gave me an understanding in a completely different way, and it made it less scary. It became easier to grasp. When you hear that they recognise what you feel... they tell you that it is completely normal to feel like this. You then understand why you feel it’. (P16)

Receiving peer support helped participants feel that they belonged to a group; they were not alone. This helped them fight the feeling of being an outsider. Acceptance and belonging were important for participants and seemed to offer them a sense of hope.

### Opportunity for nurturance

Meeting a peer supporter allowed the participants to be mutually supportive by sharing their experiences and concerns.

#### Activated an opportunity for mutual support

The participants expressed that meeting a peer supporter offered an opportunity to receive support and, at the same time, render support through the sharing of recognisable experiences and emotions. This supports the notion that conversations at the emotional level promote mutual support, as they have overlapping roles with mutual influence:

‘It is good to have someone to relate to who has some of the same struggles. The help often goes both ways. Our conversation probably also helps peer supporters. Thus, I think it is important to be able to have someone to talk to and someone to share it with, so you do not sit in this dark pit alone. Because it is a scary place to be in’. (P16)

The participants believed that sharing their personal stories and coping strategies stimulated mutual learning. Despite the peer supporter being in an explicit helper position, the peer meeting provided an opportunity for mutual support:

‘You know, we are learning from each other’. (P8)

Further, peer support activated a wish to support others and replicate the positive experience of meeting a peer supporter.

‘When I have the time, I go and meet them. I want to meet them and talk to them. There are probably some who have the same questions as me when meeting a peer supporter for the first time. I can imagine that someone newly diagnosed with HIV will need the same help. Therefore, I think it is wise to be together. Support each other’. (P10)

### Reassurance of worth

Several participants expressed how the peer supporters made them feel normal, strengthening their belief that their personal worth remained unchanged even after their HIV diagnosis.

#### Means to re-establish belief in one’s own worth

Some participants expressed ambiguity regarding their worth upon getting HIV, and how living with HIV affected their self-evaluation. Peer supporters seemed to provide an opportunity to discuss their emotions related to self-worth and acceptance:

‘There are many times I feel I do not deserve to be as healthy as I am now. However, at the same time, you need to talk to the people who understand you. It is hard to accept. I have accepted a lot in my life. I have a diagnosis. I have some bad days, and then, it is good to be able to talk about everything, right; it is not just about the HIV diagnosis, but about everything’. (P6)

Meeting a peer supporter who normalised their experiences helped the participants feel valued and less atypical. In addition, being treated as ‘any other person’ strengthened their self-worth:

‘It is important that I am part of society. I need to be part of a network in Norway. To have a normal life without people pointing out that I have HIV and should thus not come near me. Therefore, I choose not to tell people outside the hospital. When I come here, I feel normal; it is like therapy. That is important to me. I want people to treat me as normal and not be afraid’. (P1)

### Guidance

Peer supporters provided positive affirmation and advice to participants on managing their daily lives with HIV.

#### Perceived a positive affirmation of disease management

From the participants’ perspective, peer supporters provided support by sharing their own and confirming participants’ experiences, thereby contributing to improved disease management. In addition, perceiving positive affirmations from peer supporters for managing their lives with HIV was crucial for the participants:

‘They tell me stuff I probably want to know if I knew what to ask. We might have different causes, but at least we know. We are still the same in taking medications. We have common experiences and questions. So, that is what I needed, because I do not want to search for my questions online’. (P4)

Obtaining information from experience was highlighted as necessary for the participants, although all of them confirmed receiving the same information from HPs. The same information became more credible when confirmed by peer supporters. They described this as life-affirming:

‘They say you can live a good life with HIV; you just have to take medication. Life is not over. The doctor has told me several times that you do not have to believe that you will die right away. However, this is not understood inside here (pointing to the head and heart). I believed the doctor came to my house and gave me a death certificate. I had a very nice doctor, but I believed nothing of what he said. However, when I got to talk to someone living with HIV, I realised that it worked. Then, I remembered all the information I got from the healthcare professionals after meeting others with HIV’. (P8)

#### Facilitating dialogue about disease management

The participants received advice from the peer supporters about having a healthy lifestyle, specifically important for PLHIV to prevent non-communicable diseases:

‘We also talked about the importance of diet. You are especially vulnerable. Learning about what you can do in everyday life is related to exercise and diet, like regular life habits. The importance of taking medicine regularly is an important topic’. (P7)

Peer supporters facilitated dialogue related to disease management. Mutual experiences gave rise to questions and led to conversations:

‘It was nice because you have so many questions. At first, when I got the diagnosis, I thought, oh, I have to move to Berlin, because it is probably only at the sex clubs that I can get sex. You are terrified, but then you get to talk to others with HIV who have a girlfriend, for example, saying that you cannot infect others when you are taking your medication. For me, it probably helped the most to just talk to someone who has HIV’. (P9)

Some participants found it easier to direct personal questions to peer supporters than to the HPs. Thus, peer support created an opportunity to discuss health issues:

‘I need to discuss about how they cope with depression and what are their plans of disclosure; do they have to tell everyone or do they have to be open about it, or not, because you know… me coming out that I have HIV… I ask myself whether I need to. I have survived being quiet for eight years’. (P4)

### OPCs as the setting for peer support

The participants explained that they appreciated the peer support being organised at the OPCs, mainly because they experienced the latter as a safe place. In addition, they valued OPCs as a natural starting point for flexible, individualised peer support.

#### A safe place

The participants’ non-disclosure behaviour hindered them from meeting other PLHIV outside the OPCs; they had no one to talk to about HIV. When participants expressed a need to talk about HIV, the HPs became their peer support facilitators:

‘Yes, in the beginning, I felt I had to talk about HIV and meet others. It was perfect. I met with peer supporters three weeks later. It was great’. (P9)

Although some concerns were expressed regarding the personal acceptance of having HIV and not being ready to meet a peer supporter, the HPs helped them overcome these concerns:

‘I was not sure if I wanted to meet others because I struggled to accept that I had HIV. I did not want to have it; I just wanted to keep it secret. But now, I do not care. I have HIV, and there are several others living with HIV too’. (P10)

Most of the participants claimed to be afraid of disclosure if the meeting with a peer supporter happened informally, outside the OPCs. Therefore, the hospital was the only place where they wanted to meet someone in relation to their HIV status, in order to ensure that their confidentiality was maintained.

‘Like I told the nurse, you have to be discreet all the time. I survive by being quiet about this. However, although it was a time when I needed someone to talk to, I am happy that we had this peer talk here at the hospital. It is a typical problem that you really have to talk to someone, but you cannot talk about it because they probably cannot relate, and they might be stigmatising; they might be feeling weird that I have this kind of illness’. (P4)

The participants’ non-disclosure behaviour contributed to the OPCs being the only safe place in the sense that they offered a neutral, non-judgmental environment, where they knew their HIV diagnosis would be treated confidentially.

‘The hospital is experienced as a safe environment for all involved because it is a place. I think that this is important. You get to talk in peace. If you meet at a cafe, you cannot be as open or honest. Sitting in a closed room makes it much easier to share feelings. Therefore, offering an HIV-infected person to meet a peer can be valuable because you can avoid ending up in the dark as I did all alone, without anyone to talk to’. (P16)

#### A setting for flexible, individualised support

Small communities and geographical distances were expressed as factors that decrease the chance of getting the support participants needed outside the OPCs. The limited opportunity to meet peers was also related to the participants’ non-disclosure behaviour and the need for confidentiality. Hence, OPCs were the only places where they could be introduced to a peer supporter:

‘Of course, it is a challenge to gather PLHIV. It is a small town; it is too small’. (P3)

Consequently, the participants suggested that the experienced flexibility in content, time, and place for peer support positively contributed to OPC services. However, they found it crucial to adjust peer support according to individual preferences when providing support:

‘It depends on how secretive each individual is. The HPs and peer supporters ask if you want to meet someone at the hospital or if you want to meet in the city. I think it should be a flexible service based on each individual. To begin with, I think it is important that you meet a peer supporter together with the healthcare professionals. Further, everyone has been asked what they prefer’. (P5)

## Discussion

This study aimed to explore how PLHIV experienced meeting peer supporters in an OPC. To our knowledge, this is the first study investigating experiences of PLHIV with peer support in OPCs in a Scandinavian, low-prevalence, high-income country.

This study demonstrates that, in terms of peer support, each of Weiss’ six provisions of social relationships [35] is affirmed through our findings, except for the provision of a reliable alliance. Our results suggest that the participating service users do not express to need a peer supporter to be motivated for regular care or to identify local resources. This might be the case because the service users are already connected to the OPCs, and the peer support services thereby shoulder the already existing services. Therefore, based on our results, we could argue that peer support complementary with the OPCs’ existing services, provides a diversity of individualised support responsive to the receivers’ personal needs and preferences [23, 26]. Furthermore, this individualised support is in accordance with the WHO’s strategy regarding integrated and person-centred chronic care to promote well-being for PLHIV [6, 27, 28].

The study revealed differences among the participants regarding how they experienced the content of the peer support. In the present study, several participants lacked emotionally close relationships in their everyday lives, or had their former close relationships negatively affected upon getting diagnosed with HIV. In fact, previous studies show that social isolation is often related to HIV, which diminishes support [52]. This is despite our knowledge that expressing personal emotions through social support can increase people’s resilience to stigma [20, 53, 54]. PLHIV with non-disclosure behaviour and few or no close relations have been found to become emotionally attached to peer supporters. Many PLHIV are immigrants who have not disclosed their diagnosis due to a fear of stigma [13, 52], suggesting potential inequalities in health within the population of PLHIV in Norway. Our results, which are in line with other studies [17, 53], demonstrate that until stigma connected to HIV is reduced globally, both disclosure behaviour and social support for PLHIV in Norway can be compromised. Thus, the results suggest the need for equitable, individualised peer support, as a complement to existing healthcare services, to increase the emotional well-being of PLHIV [3, 26, 28].

Peer support provided participants in this study with a sense of belonging to a group with similar experiences and concerns, without any fear of rejection, which was not found elsewhere, following Weiss’ [35] description of common-concern relationships. Baumeister and Leary [55] described the anxiety arising from imagined or expected social rejection, which could be seen in the non-disclosure behaviours of PLHIV mentioned by our participants. Similar to previous findings [56], several participants’ non-disclosure of HIV increased their feeling of loneliness. Past literature supports the contribution of peer supporters in terms of just ‘being there’, to be of substantial value for the participants [31], as corroborated in our study. This sense of belonging strengthened their belief in their worth, alleviating the internalised stigma associated with HIV [52].

Our results align with previous findings that mutual support between peers increases participants’ sense of belonging [30, 55]. As affirmed by our study, human beings are driven to form and maintain positive interpersonal relationships in which mutual care is perceived [55]. Further, the dialogue between peers concerning mutual experiences was perceived as positive affirmation and advice on living positively with HIV, consistent with one of the known key functions of peer support described in *Peers in Progress* [23, 31].

Norway is a low-prevalence country [57] and has achieved the UNAIDS 90–90-90 target [65,44]. Yet, perhaps partially because of this situation, PLHIV in Norway experience loneliness, which seems to be linked to the lack of spaces where living with HIV is regarded as ‘natural and unproblematic’. This is doubly problematic, as informal peer support is challenging in Norway, given the significant geographical distances and the anticipated intersectional stigma among PLHIV [5, 13]3. Even though telehealth is expected to play a greater role in future global healthcare services, peer support is not yet available for PLHIV in Norway as a part of the telehealth services. Overall, this affects their quality of life and well-being [56]. A person-centred approach highlights the importance of contextual factors, which is evident in our research. The participants appreciated that peer support was organised and located at the OPCs because they provided a safe environment where confidentiality was guaranteed. In addition, peer supporters, as a part of the OPC services, allowed for enhanced equal access to peers. Therefore, incorporating peer supporters as a part of OPC services might increase the opportunity to provide flexible, individualised support to every individual living with HIV. These findings complement the Global Health Sector Strategy on HIV 2016–2020 [6], emphasising the value of HIV services that are adjusted for various populations and locations.

### Relevance to clinical practice

Addressing the evolving needs of PLHIV is vital to achieving and maintaining good health-related quality of life; peer support acts as a contribution to the same. Thus, this study adds to our knowledge and understanding of the complex needs of PLHIV, calling for a holistic approach to ensure well-being [28]. In today’s HIV treatment landscape, the continuum of care goes far beyond virologic suppression, with innovations such as digital technologies becoming important facilitators of health for responding to the growing needs of PLHIV [56]. However, this study also highlights the importance of face-to-face peer support as part of a continuing, flexible, and individualised support to strengthen the well-being of PLHIV. In addition, studies indicate that personalised peer support with routine medical care is superior to a routine clinic follow-up in improving the health outcomes of PLHIV [25]. Concerning implications, to enhance the quality of life of PLHIV, this study provides valuable knowledge of peer support as a lower-threshold intervention to meet daily emotional needs. Furthermore, it contributes to an increased awareness of the additional assistance a peer supporter provides in shouldering the existing healthcare services, as supported by other reviews [25, 58] in response to the intersecting challenges facing PLHIV [59]. The study also highlights the necessity of embedding peer support in OPCs to equalise peer support opportunities for PLHIV, overcoming barriers in contacting non-governmental organisations in a culture of non-disclosure. Furthermore, most non-governmental organisations are connected to religious organisations. Therefore, from the service users' point of view, most non-governmental organisations represent values not aligned with being a Muslim or a gay man. The OPCs also represent the only place all people diagnosed with HIV have follow-up care, whilst non-governmental organisations are only present in larger cities. Expanded telehealth services might provide PLHIV with peer support offered by either the OPCs or the non-governmental organisations, suggesting a more individualised approach to decrease peer support barriers for future practice [60].

### Strength and limitations of the study

This study was based on 16 participants who spent various amounts of time with their peer supporters. In addition, some of the interviews were conducted in English when the participants requested it, based on their limited competence in the Norwegian spoken language. Although there was diversity among both peer supporters and participants in the current study in terms of age, sexual orientation, time since diagnosis, and country of origin, we did not have the resources to interview PLHIV who could not communicate in Norwegian or English. Overall, this may have affected the participants’ shared experiences and reflections. Nonetheless, the results highlighted multiple experiences of PLHIV with the provision of peer support.

Additionally, the participants were recruited by HPs. This could have affected their decision to participate, although the HPs already had an established relationship with the participants. The participants were informed that their decision would not negatively impact the HIV care they received. Every step in the analysis was discussed with all the authors and the advisory group to ensure credibility.

## Conclusions

This study highlighted the content of peer support from the receiver’s perspective in the context of OPCs. The participants’ experiences aligned with previous findings, with peer support contributing to mutual emotional support between peers. This is particularly important in cultures of non-disclosure where PLHIV experience intersectional stigma. Additionally, the results of this study emphasised the OPCs as supportive surroundings for facilitating peer support, ensuring confidentiality in peer support outreach. Thereby, peer support was found to positively contribute to individualising OPC services to meet the changing needs of PLHIV.

## Supplementary Information

**Additional file 1.**

**Additional file 2.**

**Additional file 3.**

## Data Availability

The datasets used in this study are presented in this article. Approval from the NSD and the participants were only linked to this study. Further information is available from the corresponding author upon request.

## Abbreviations

HIV: Human immunodeficiency virus
PLHIV: People living with HIV
ART: Antiretroviral therapy
CLLC: Chronic lifelong condition
OPCs: Outpatient clinics
HPs: Healthcare professionals
WHO: World Health Organization
UNAIDS: United Nations Programme on HIV and AIDS

## References

[1] Lohse N, Obel N (2016). Update of survival for persons with HIV infection in Denmark. Ann Intern Med.

[2] WHO. Fact Sheet 2021: Number of people (all ages) living with HIV Estimates by WHO region 2020. World Health Organization; 2021. Cited August 2021 [Internet]. https://www.who.int/news-room/fact-sheets/detail/hiv-aids.

[3] WHO (2019). Maintaining and improving quality of care within HIV clinical services.

[4] Parcesepe AM, Bernard C, Agler R, Ross J, Yotebieng M, Bass J (2018). Mental health and HIV: research priorities related to the implementation and scale up of 'treat all' in sub-Saharan Africa. J Virus Erad.

[5] Brandt R (2009). The mental health of people living with HIV/AIDS in Africa: a systematic review. Afr J AIDS Res.

[6] WHO. Global health sector strategy on HIV: 2016–21; 2016. p. 10–55. https://www.who.int/hiv/strategy2016-2021/ghss-hiv/en/.

[7] Engelhard EAN, Smit C, van Dijk PR, Kuijper TM, Wermeling PR, Weel AE (2018). Health-related quality of life of people with HIV: An assessment of patient related factors and comparison with other chronic diseases. AIDS.

[8] Bristowe K, Clift P, James R, Josh J, Platt M, Whetham J (2019). Towards person-centred care for people living with HIV: What core outcomes matter, and how might we assess them? a cross-national multi-centre qualitative study with key stakeholders. HIV Med.

[9] Relf MV, Holzemer LW, holt L, Nyblade L, Ellis Caiola C (2021). A review of the state of the science of HIV and Stigma: context, conceptualisation, measurement, interventions, gaps, and future priorities. J Assoc Nurs AIDS Care.

[10] Berg RC, Ross MW (2014). The second closet: a qualitative study of HIV stigma Among seropositive gay men in a Southern U. S. City. Int J Sex Health..

[11] Chaudoir SR, Fisher JD. Stigma and the “Social Epidemic” of HIV: Understanding Bidirectional Mechanisms of Risk and Resilience. Oxford Library of Psychology. 1 ed. Oxford University Press; 2018. p. 1–34.

[12] Pantelic M, Steinert JI, Park J, Mellors S, Murau F (2019). ‘Management of a spoiled identity’: systematic review of interventions to address self-stigma among people living with and affected by HIV. BMJ Glob Health..

[13] Major B, Schmader T. Stigma, Social Identity Threat, and Health. Oxford Library of Psychology. 1 ed. Oxford University Press; 2018. p. 85–103.

[14] Liamputtong P. Stigma, Discrimination and Living with HIV/AIDS: A Cross-Cultural Perspective. 2013 ed. Dordrecht: Springer Netherlands; 2013. p. 1–118.

[15] Hatzenbuehler ML, Phelan JC, Link BG (2013). Stigma as a fundamental cause of population health inequalities. Am J Public Health.

[16] Sokol R, Fisher E (2016). Peer support for the hardly reached: a systematic review. Am J Public Health.

[17] Smith R, Rossetto K, Peterson BL (2008). A meta-analysis of disclosure of one's HIV-positive status, stigma and social support. AIDS Care.

[18] Earnshaw VA, Lang SM, Lippitt M, Jin H, Chaudoir SR (2015). HIV stigma and physical health symptoms: do social support, adaptive coping, and/or identity centrality act as resilience resources?. AIDS Behav.

[19] Dulin AJ, Dale SK, Earnshaw VA, Fava JL, Mugavero MJ, Napravnik S (2018). Resilience and HIV: a review of the definition and study of resilience. AIDS Care.

[20] Garrido-Hernansaiz H, Alonso-Tapia J (2017). Social support in newly diagnosed people living With HIV: expectations and satisfaction along time, predictors, and mental health correlates. J Assoc Nurses AIDS Care.

[21] Positively UK. National Standards for Peer Support in HIV. 2016. p. 1–35. http://hivpeersupport.com/wp-content/uploads/2017/08/national_standards_final_web.pdf.

[22] Dunbar W, Labat A, Raccurt C, Sohler N, Pape JW, Maulet N (2020). A realist systematic review of stigma reduction interventions for HIV prevention and care continuum outcomes among men who have sex with men. Int J STD AIDS.

[23] Fisher EB. Global Evidence for Peer Support: Humanizing health care. Peer for Progress; 2014; p. 1–44. http://peersforprogress.org/wp-content/uploads/2014/09/140911-global-evidence-for-peer-support-humanizing-health-care.pdf.

[24] Simoni JM, Nelson KM, Franks JC, Yard SS, Lehavot K (2011). Are peer interventions for HIV efficacious?. A systematic review AIDS Behav.

[25] Berg RC, Page S, Øgård-Repål A (2021). The effectiveness of peer-support for people living with HIV: a systematic review and meta-analysis. PLOS ONE..

[26] McCormack, B., McCance, T. Person-centred practice in nursing and health care: theory and practice. Second edition. ed. Chichester: Wiley-Blackwell; 2017. p. 13–36.

[27] WHO. People-centred and integrated health services: an overview of the evidence: interim report. Geneva: World Health Organization; 2015. p. 1–18.

[28] Lazarus JV, Safreed-Harmon K, Barton SE, Costagliola D, Dedes N, del Amo Valero J (2016). Beyond viral suppression of HIV - the new quality of life frontier. BMC Med..

[29] World Health O (2019). One-to-one peer support by and for people with lived experience: WHO QualityRights guidance module.

[30] MacLellan J, Surey J, Abubakar I, Stagg HR (2015). Peer support workers in health: a qualitative metasynthesis of their experiences. PLOS ONE..

[31] Fisher EB, Tang PY, Coufal MM, Liu Y, Jia W, Daaleman TP, Helton MR (2018). Peer support. Chronic Illness Care: Principles and Practice.

[32] Genberg BL, Shangani S, Sabatino K, Rachlis B, Wachira J, Braitstein P (2016). Improving engagement in the HIV care cascade: a systematic review of interventions involving people living with HIV/AIDS as peers. AIDS Behav.

[33] Øgård-Repål A, Berg RC, Fossum M (2021). A scoping review of the empirical literature on peer support for people living with HIV. Journal of the International Association of Providers of AIDS Care.

[34] Swarbrick.  (2006). A wellness approach. Psychiatr Rehabil J.

[35] Weiss R, Rubin Z (1974). The provisions of social relationships. Doing unto Others.

[36] Borkman. Understanding Self-help/Mutual Aid : Experiential Learning in the Commons. New Brunswick: Rutgers University Press. 1999. p. 24–50.

[37] Cutrona C, Russell D (1987). The provisions of social relationships and adaptation to stress. Adv Personal Relat.

[38] Assarroudi A, HeshmatiNabavi F, Armat MR, Ebadi A, Vaismoradi M (2018). Directed qualitative content analysis: The description and elaboration of its underpinning methods and data analysis process. J Res Nurs.

[39] Hsieh HF, Shannon SE (2005). Three approaches to qualitative content analysis. Qual Health Res.

[40] Alvesson M, Sköldberg K. Reflexive methodology: new vistas for qualitative research. Third edition. ed. Los Angeles. California: SAGE; 2018. p. 10–157.

[41] Malterud K. Kvalitative forskningsmetoder for medisin og helsefag. 4. utg. ed. Oslo: Universitetsforl.; 2017. p. 1–254.

[42] Polit DF, Beck CT. Essentials of nursing research: appraising evidence for nursing practice. 9th ed. ed. Philadelphia: Wolters Kluwer; 2018. p. 1–442.

[43] Association NM. [Faglige retningslinjer for oppfølging og behandling av hiv; 2021] in Norwegian; 2021. p. 1–54. https://www.legeforeningen.no/foreningsledd/fagmed/norsk-forening-for-infeksjonsmedisin/aktuelt/2020/faglige-retningslinjer-for-oppfolging-og-behandling-av-hiv-2021/.

[44] Whittaker R, Case KK, Nilsen Ø, Blystad H, Cowan S, Kløvstad H, et al. Monitoring progress towards the first UNAIDS 90–90-90 target in key populations living with HIV in Norway. BMC Infect Dis. 2020;20(1):451;2–11. 10.1186/s12879-020-05178-1 PMC7318482 32590964

[45] UNAIDS. 90–90–90 An ambitious treatment target to help end the AIDS epidemic (Internet). Geneva: Switzerland; 2014.

[46] Berg RC, Gamst A, Said M, Aas KB, Songe SH, Fangen K, et al. True user involvement by people living with HIV is possible: Description of a user-driven HIV clinic in Norway. J Assoc Nurses AIDS Care. 2015;26(6):732–42. 10.1016/j.jana.2015.07.002 26255897

[47] Ministries N. Talk about it! Sexual Health Strategy (2017–2022). Minist Health Care; 2016. https://www.regjeringen.no/no/dokumenter/snakk-om-det/id2522933/.

[48] Miles MB, Huberman AM. Qualitative data analysis: an expanded sourcebook. 2nd ed. Thousand Oaks: Sage; 1994. p. 1–338.

[49] Malterud K, Siersma VD, Guassora AD (2016). Sample size in qualitative interview studies: guided by information power. Qual Health Res.

[50] Cutrona CE, Russell DW. Type of social support and specific stress: Toward a theory of optimal matching. Social support: An interactional view. Wiley S Pers Processes. Oxford: John Wiley & Sons; 1990. p. 319–66.

[51] Tong A, Sainsbury P, Craig J. Consolidated criteria for reporting qualitative research (COREQ): A 32-item checklist for interviews and focus groups. https://www.equator-network.org/reporting-guidelines/coreq/. Int J Qual Health Care. 2007;19:349–57. 10.1093/intqhc/mzm042 17872937

[52] Relf MV, Holzemer WL, Holt L, Nyblade L, Ellis Caiola C. A Review of the State of the Science of HIV and Stigma: Context, Conceptualization, Measurement, Interventions, Gaps, and Future Priorities. J Assoc Nurses AIDS Care. 2021;32:392–407. 10.1097/JNC.0000000000000237 PMC9208366 33654005

[53] Cohen S, Wills TA. Stress, social support, and the buffering hypothesis. Psychol Bull. 1985;98(2):310–57. 3901065

[54] Cohen S (1988). Psychosocial models of the role of social support in the etiology of physical disease. Health Psychol.

[55] Baumeister RF, Leary MR (1995). The need to belong: Desire for interpersonal attachments as a fundamental human motivation. Psychol Bull.

[56] Grønningsæter AB, Hansen ILS. Aging with HIV; 2018. https://www.fafo.no/zoo-publikasjoner/summaries/item/aging-with-hiv.

[57] Caugant D, Kløvstad H, Øea N. Surveillance of sexually transmitted infections. Norwegian Institute of Public Health; 2021. https://www.fhi.no/publ/2021/overvakning-av--seksuelt-overforbare-infeksjoner.-arsrapport-2020/.

[58] Dave S, Peter T, Fogarty C, Karatzas N, Belinsky N, Pant Pai N (2019). Which community-based HIV initiatives are effective in achieving UNAIDS 90–90–90 targets? A systematic review and meta-analysis of evidence (2007–2018). PLOS ONE..

[59] Meyer IH (2003). Prejudice, social stress, and mental health in lesbian, gay, and bisexual populations: Conceptual issues and research evidence. Psychol Bull.

[60] World Health Organization. Global strategy on digital health 2020–2025. World Health Organization; 2021. https://www.who.int/health-topics/digital-health#tab=tab_1. 10.2471/BLT.20.253955 PMC7133480 32284641

