# Supplementary material for: Peer support in an outpatient clinic for people living with human immunodeficiency virus: a qualitative study of service users’ experiences
Source: BMC Health Serv Res. 2022 Apr 25;22:549. doi: 10.1186/s12913-022-07958-8 (PMC9036816; doi:10.1186/s12913-022-07958-8)
Supplement: Supplementary file 1 — Additional file 1. [file 12913_2022_7958_MOESM1_ESM.docx]

# Interview guide

The purpose of this interview is to tell me as much as possible about your experiences of peer support to gain expanded knowledge. I also want to talk to you about how you were informed about peer support and how the meetings were organised. In addition, I would like to hear more about your experiences of living with HIV.

## Introduction

- Presentation of the project (theme and the contribution needed from participants)
- Practical aspects of the interview (use of time, the opportunity for flexibility if something interesting arose during the conversation)
- Research ethics related to the interviews and the bigger project. Particular focus on my duty of confidentiality and confidential treatment of the data (emphasis on openness and honesty; there are no correct answers and that the participants themselves decide what they want to share along the way.

*Names, surnames, and contact information are obtained and coded with respondents 1-4.*

To prevent the disclosure of personal information about third parties, we will discuss privacy with the informant prior to the interview. However, we want to be aware of how we ask the questions and let them know that they must use other names / refrain from using names when referring to people, omitting any characteristics related to them.

## Background information

1. Age
2. Gender
3. How long have you been living with HIV?
4. In what country were you born?
5. Sexual orientation

## **Themes**

About meeting a peer supporter

1. Tell me about your experiences with meeting a peer supporter.
   1. How was it organised?
   2. Where did you meet?
   3. How long did the meeting last?
2. What challenges did you experience when meeting a peer supporter? Is there something you believe should be improved or changed?
3. What experiences did you think a peer supporter could contribute to your daily life?
4. Can you share any experience where you realised that the peer support meeting became important or valuable?
5. What are your expectations when meeting a peer supporter?
6. What do you want to achieve when meeting a peer supporter?
7. Is there something you think is challenging about meeting a peer supporter?
8. How are the expected negative effects or challenges mentioned?
9. What is required for you to meet a peer supporter? Does it imply any costs?
10. In what way do you think meeting a peer supporter could affect your health?
11. Do you have any ethical concerns about meeting a peer supporter?
12. Do you think it is realistic (feasible) that people living with HIV can be offered to meet a counterpart?
13. Is there anything you want to add / do you have any further comments?

Your experiences related to social support and stigma

1. To what extent and how do you experience support from your surroundings regarding your HIV diagnosis?
2. Can you come up with a situation that describes your need for support?
3. Have there been periods or situations where you experienced a greater degree of uncertainty and predictability related to your situation?
4. Have you experienced periods of lack of control?
5. Can you come up with a situation that describes how social support has been helpful?
6. What expectations do you have for your surroundings related to your diagnosis?
7. Have you been exposed to, or have you heard of others with the same diagnosis experiencing any form of discrimination or stigma?
8. If you have experienced this situation, can you tell us about it?
