# Supplementary material for: Peer support in an outpatient clinic for people living with human immunodeficiency virus: a qualitative study of service users’ experiences
Source: BMC Health Serv Res. 2022 Apr 25;22:549. doi: 10.1186/s12913-022-07958-8 (PMC9036816; doi:10.1186/s12913-022-07958-8)
Supplement: Supplementary file 2 — Additional file 2. [file 12913_2022_7958_MOESM2_ESM.docx]

Data analysis process according to Assarroudi et al. [36]

| *Phase* | *Steps* | *Description* |
| --- | --- | --- |
| Preparation | Acquiring the necessary general skills | The first author familiarised herself with the concept of social support, stigma, and existing peer support programs for people living with HIV through a review of available research and the current body of knowledge. |
|  | Selecting the appropriate sampling strategy | Participants were purposively recruited from outpatient clinics, with variations in their sociodemographic characteristics. Saturation was reached; all participants willing to participate in the study were recruited based on the study’s purpose. |
|  | Deciding on the analysis of manifest and/or latent content | To address the study aim, both manifest and latent content was analysed to gain a deeper understanding. |
|  | Developing an interview guide | The interview guide was developed with the advisory group, containing semi-structured, open-ended questions based on previous research and the current study’s aims [34,35]. We asked what the participant thought about peer support and their thoughts related to stigma and social support in general. |
|  | Conducting and transcribing interviews | The interviewer was provided with an interview guide for the session. Interviews were transcribed verbatim by the first author. |
|  | Specifying the unit of analysis | The transcribed data were used as the unit of analysis. |
|  | Immersion in data | During the coding process, the transcribed interviews were read several times while listening to the recordings, ensuring that all latent content was captured, differentiating between speaker and context. |
| Organisation | Developing a formative categorisation matrix | The pre-determined categories were derived from previous research [34,35]. Then, potential sub-categories were identified through an inductive approach [37]. |
|  | Theoretically defining themes and sub-themes | The definition of each category was checked for accuracy and objectiveness based on the existing body of knowledge [23] and theories [34,35]. |
|  | Determining coding rules for themes | Rules were created for themes to ensure their trustworthiness. Following these rules informed the coder of the clear distinction between the categories. |
|  | Pre-testing the categorisation matrix | Two researchers coded the interviews. Each tested the categorisation matrix independently and they discussed challenges in using the matrix. This was repeated after coding more interviews as new categories emerged, facilitating the refinement of categories and increasing inter-coder reliability and the study’s trustworthiness. |
|  | Choosing and specifying the anchor samples for each theme | Explicit and concise sample quotes were selected for each theme. |
|  | Performing the main data analysis | Meaningful units were derived based on the study’s aim and categorisation matrix, and summarised into codes. The advisory group was involved in the coding process. |
|  | Inductive abstraction of themes from preliminary codes | The preliminary and emerging codes were grouped based on their similarities, resulting in ‘generic categories’ for this study. This grouping was conducted with the advisory group. |
|  | Establishment of links between generic categories and themes | The conceptual and logical links were created through constant comparison of the generic categories and themes throughout the coding process, facilitating the nesting of the generic categories into new or pre-existing categories. |
| Reporting | Reporting all steps of directed content analysis and findings | A detailed report of the findings is reported in the Results section. |
