# Supplementary material for: Peer support in an outpatient clinic for people living with human immunodeficiency virus: a qualitative study of service users’ experiences
Source: BMC Health Serv Res. 2022 Apr 25;22:549. doi: 10.1186/s12913-022-07958-8 (PMC9036816; doi:10.1186/s12913-022-07958-8)
Supplement: Supplementary file 3 — Additional file 3. [file 12913_2022_7958_MOESM3_ESM.docx]

Definition of categories, operationalisation, and the meaning of each provision from the perspective of peer support

| *Category* | *Definition* | *Operationalisation* | *Examples of participants’ narratives* |
| --- | --- | --- | --- |
| Attachment | Emotional closeness from which one derives a sense of security. | Receiving emotional support from people living with HIV when needed | ‘I got support here at the hospital, and this is like my ‘health family’, talking to the nurse and the peer supporters. That is important’. (Cries when saying this) (P1) |
| Social integration | A sense of belonging to a group that shares similar interests, concerns, and recreational activities | Receiving encouragement, sharing experiences, and helping people deal with potential stress related to living with HIV | ‘It was good. I am not alone. I knew I was not alone, but I knew no one else. So really, meeting someone was ...’ (P3) |
| Opportunity for nurturance | The sense that others rely upon one for their well-being | Mutual support in the sense that you help others through sharing personal experiences of living with HIV | ‘It is good to have someone to relate to who has some of the same struggles. The help often goes both ways. Our conversation probably also helps peer supporters’. (P16) |
| Reassurance of worth | Recognition of one’s competence, skills, and value by others | Helping people living with HIV understand that the diagnosis does not affect their value as a person | ‘There are many times I feel I do not deserve to be as healthy as I am now. However, at the same time, you need to talk to the people who understand you. It is hard to accept. I have accepted a lot in my life. I have a diagnosis. I have some bad days, and then, it is good to be able to talk about everything, right; it is not just about the HIV diagnosis, but about everything’. (P6) |
| Reliable alliance (Practical help) | The assurance that others can be counted upon for tangible assistance | Serving as a liaison between patients and clinical care, motivating patients to communicate and assert themselves to obtain regular and quality care, helping to identify local resources when needed |  |
| Guidance | Advice and information | Helping people living with HIV apply disease management in their daily lives | ‘We also talked about the importance of diet. You are especially vulnerable. Learning about what you can do in everyday life is related to exercise and diet, like regular life habits. The importance of taking medicine regularly is an important topic’. (P7) |
| Program-related factors | Factors related to peer support situated in outpatient clinics | Aspects concerning the suitability of locating peer support for people living with HIV in outpatient clinics | ‘The hospital is experienced as a safe environment for all involved because it is a neutral place. I think that this is important. You get to talk in peace. If you meet at a cafe, you cannot be as open or honest. Sitting in a closed room makes it much easier to share your feelings. Therefore, offering an HIV-infected person to meet a peer can be valuable because you can avoid ending up in the dark as I did all alone, without anyone to talk to’. (P16) |
